# Emerging therapies for smoke inhalation injury: a review

**DOI:** 10.1186/s12967-020-02300-4

**Published:** 2020-03-30

**Authors:** Alexandra Mercel, Nick D. Tsihlis, Rob Maile, Melina R. Kibbe

**Affiliations:** 1 Department of Surgery, University of North Carolina at Chapel Hill, 4041 Burnett Womack, 101 Manning Drive, CB# 7050, Chapel Hill, NC 27599-7050 USA; 2 Department of Biomedical Engineering, University of North Carolina at Chapel Hill, Chapel Hill, USA; 3 Department of Microbiology and Immunology, University of North Carolina at Chapel Hill, Chapel Hill, USA

**Keywords:** Burn inhalation injury, Emerging therapy, Smoke inhalation injury, Review

## Abstract

**Background:**

Smoke inhalation injury increases overall burn mortality by up to 20 times. Current therapy remains supportive with a failure to identify an optimal or targeted treatment protocol for smoke inhalation injury. The goal of this review is to describe emerging therapies that are being developed to treat the pulmonary pathology induced by smoke inhalation injury with or without concurrent burn injury.

**Main body:**

A comprehensive literature search was performed using PubMed (1995–present) for therapies not approved by the U.S. Food and Drug Administration (FDA) for smoke inhalation injury with or without concurrent burn injury. Therapies were divided based on therapeutic strategy. Models included inhalation alone with or without concurrent burn injury. Specific animal model, mechanism of action of medication, route of administration, therapeutic benefit, safety, mortality benefit, and efficacy were reviewed. Multiple potential therapies for smoke inhalation injury with or without burn injury are currently under investigation. These include stem cell therapy, anticoagulation therapy, selectin inhibition, inflammatory pathway modulation, superoxide and peroxynitrite decomposition, selective nitric oxide synthase inhibition, hydrogen sulfide, HMG-CoA reductase inhibition, proton pump inhibition, and targeted nanotherapies. While each of these approaches shows a potential therapeutic benefit to treating inhalation injury in animal models, further research including mortality benefit is needed to ensure safety and efficacy in humans.

**Conclusions:**

Multiple novel therapies currently under active investigation to treat smoke inhalation injury show promising results. Much research remains to be conducted before these emerging therapies can be translated to the clinical arena.

## Background

Inhalation injury is a devastating injury that occurs in up to one-third of all burn patients and contributes to an increase in overall mortality. The addition of smoke inhalation injury to burn injury may contribute to an increase in mortality up to 20 times higher than seen in burn injury alone [1–3]. Although it most commonly occurs after exposure to fire, inhalation injury can occur after exposure to chemicals, fumes, gases, vapors, or smoke. Smoke inhalation causes pulmonary injury through direct heat exposure, or, more commonly, through exposure to chemicals of combustion and their subsequent local and systemic effects [4].

Despite the significant damage that results from inhalation injury, current therapy remains supportive and includes bronchodilators, muscarinic antagonists, and mucolytics such as N-acetyl cysteine and aerosolized heparin. These medications are most commonly administered in combination with other supportive means including bronchiolar lavage and mechanical ventilation, but no specific approved targeted therapy has been identified [5, 6].

The purpose of this review is to provide a synopsis of emerging therapies being developed for the treatment of inhalation injury that are not yet approved by the FDA. This review summarizes therapies for inhalation injury with or without concurrent burn injury. Therapeutic modalities include stem cell therapy, anticoagulation therapy, selectin inhibition, inflammatory pathway modulation, superoxide and peroxynitrite decomposition, selective nitric oxide synthase (NOS) inhibition, hydrogen sulfide, HMG-CoA reductase inhibition, proton pump inhibition, and targeted nanotherapies. While these therapeutic approaches have been evaluated in a variety of early-stage animal models, further study will be required before progressing to human studies.

### Pathophysiology

The pathophysiology of inhalation injury varies depending on anatomical location and is classified as either upper airway thermal injury, lower airway chemical injury, or lung parenchymal and systemic injury [5, 6]. Upper airway injury, although rare, is associated with heat transfer resulting in thermal burns and can be associated with severe injury [2]. Lower airway injury occurs secondary to the chemical combustion of smoke, which releases byproducts that result in increased bronchial blood flow, loss of pulmonary hypoxic vasoconstriction, airway edema, cast formation with airway obstruction, complement activation, release of reactive oxygen species, increased oxidative stress, and activation of pro-inflammatory immune cells and cytokines [1, 2]. Lung tissue damage, or parenchymal injury, results in impaired oxygen and carbon dioxide exchange, inhibited pulmonary mechanics, and an overall decline in the ratio of arterial partial pressure to fractional inspired oxygen (PaO_2_/FiO_2_) [2, 6].

## Main text

We performed a comprehensive literature search with PubMed to identify emerging therapies for smoke inhalation injury with or without concurrent burn injury. We searched for therapeutics that are still under investigation and not yet approved by the U.S. Food and Drug Administration (FDA). We used the English language and searched for articles using key words *inhalation, airway, smoke, lung,* and *pulmonary* in isolation or in combination with *treatment, therapy, burn, smoke, emerging,* and *thermal.* We acknowledge that investigator bias may have affected our investigative approach. To limit this bias and ensure a comprehensive review, we noted all additional sources that were referenced in publications during our initial search and, if applicable, included them in our review.

Emerging therapies were divided into categories based on their therapeutic strategy: stem cell therapy, anticoagulation therapy, selectin inhibition, inflammatory pathway modulation, superoxide and peroxynitrite decomposition, selective NOS inhibition, hydrogen sulfide, HMG-CoA reductase inhibition, proton pump inhibition, and targeted nanotherapies (Table 1). Models include either smoke inhalation alone or burn injury plus smoke inhalation injury. Individual therapies were each assessed based on their method of administration, safety profile, systemic effects, effect on pulmonary pathophysiology, and overall efficacy and mortality (if reported).

**Table 1:** Emerging therapies for smoke inhalation injury

Therapeutic strategy	Type of therapeutic	Model	Animal	Route of administration	Results
Stem cell	Bone marrow derived mesenchymal stem cells	Smoke inhalation Smoke inhalation Smoke inhalation Smoke inhalation Smoke inhalation	Rabbit [7]	IV, marginal ear vein	Decreased VEGF Decreased total lung water content
			Rabbit [14, 15]	IV, marginal ear vein	Decreased pro-inflammatory cytokines in serum, increased anti-inflammatory cytokines in serum Improved histopathology Decreased wet-to-dry ratio
			Rat [12]	IV, tail vein	Decreased wet-to-dry ratio Decreased IL-8 Increased IL-10
			Rat [13]	IV, tail vein	Decreased wet-to-dry ratio Improved histopathology
			Mouse [16]	IV, tail vein	Decreased levels of TNF-alpha Increased migration of stem cells to lung tissue in injured mice
	Human amnion mesenchymal stem cells	Smoke inhalation	Rat [18]	IV, tail vein	Decreased wet-to-dry ratio Improved histopathology Improved oxygenation Increased surfactant levels
	Adipose derived mesenchymal stem cells	Smoke inhalation	Sheep [19]	IV, central venous infusion	Decreased pulmonary vascular permeability Decreased wet-to-dry ratio Improved oxygenation
Anticoagulants	Tissue plasminogen activator	Burn and smoke inhalation	Sheep [23]	Aerosolized	Improved airway obstruction Decreased wet-to-dry ration Improved vascular leakage
	Antithrombin III/heparin	Burn and smoke inhalation	Sheep [24] Sheep [25]	Combined aerosolized IV infusion- ATIII Aerosolized- heparin	Improved airway obstruction Improved pulmonary mechanics and oxygenation Decreased wet-to-dry ratio
Selectin inhibition	P selectin	Burn and smoke inhalation	Sheep [26]	IV injection	No pulmonary protection in injury vs. control
	L selectin	Burn and smoke inhalation	Sheep [28]	IV injection before injury	Improved microvascular permeability No significant improvement in oxygenation
			Sheep [27]	IV injection after injury	Decreased systemic neutrophil infiltration Improved vascular permeability Decreased pulmonary edema
Immunomodulation	CXCL-1 neutralization	Burn and smoke inhalation	Mouse [30]	IV, tail vein	Improved lung histopathology Decreased wet-to-dry ratio Decreased pro inflammatory cytokines Decreased pulmonary neutrophil infiltration
	Puerarin	Smoke inhalation	Rat [31]	IP injection	Improved lung histopathology Decreased neutrophil infiltration Decreased pulmonary vascular permeability
	Perfluorohexane	Burn and smoke inhalation	Human [32] (RCT)	Intratracheal instillation	Improved pulmonary mechanics and oxygenation Decreased neutrophil infiltration Decreased pro inflammatory cytokines
	SOCS-1	Smoke inhalation	Mouse [37]	Intratracheal instillation	Improved mortality Improved lung histopathology Decreased pro inflammatory cytokines
	Glutamine	Smoke inhalation	Rat [44]	IV, tail vein	Decreased pulmonary edema Decreased pro inflammatory cytokines Improved histopathology Decreased fibrosis Increased levels of protective heat shock proteins
Recombinant superoxide dismutase	Manganese superoxide dismutase	Smoke inhalation Smoke inhalation	Sheep [51]	IV bolus	No significant change in oxygenation or lung lymph flow
			Sheep [52]	Aerosolized	No significant change in oxygenation or wet-to-dry ratio Decreased conjugated dienes
Peroxynitrite decomposition catalyst	W-85	Burn and smoke inhalation	Sheep [54]	Intra-arterial injection, bronchial artery	Improved pulmonary oxygenation Decreased pulmonary vascular permeability
	INO-4885	Burn and smoke inhalation	Sheep [55]	IV bolus followed by infusion	Improved oxygenation and pulmonary mechanics Decreased pulmonary edema Decreased pro inflammatory cytokines Decreased VEGF, PARP
	R-100	Smoke inhalation, bacterial injury	Sheep [56]	IV bolus followed by infusion	Improved oxygenation and pulmonary mechanics No change in histopathology or wet-to-dry ratio
iNOS inhibition	MEG	Burn and smoke inhalation	Sheep [87]	IV infusion	Increased iNOS levels in treatment groups Decreased pulmonary edema Improved pulmonary vascular permeability
	BBS-2 (48 h)	Burn and smoke inhalation	Sheep [5Improved lung histopathology Decreased ROS, lipid peroxidation, acetylcholine esterase activity 7]	IV infusion, 48 h	Improved oxygenation and pulmonary mechanics Decreased pulmonary shunt fraction Improved lung lymph flow Decreased pulmonary edema Improved airway obstruction
	BBS-2 (24 h)	Burn and smoke inhalation	Sheep [48]	IV infusion, 24 h	Improved pulmonary gas exchange Improved airway mechanics Decreased pulmonary edema
	BME	Smoke inhalation	Rat [58]	Oral	Decreased levels of nitrite, nitrate, PARP, NF-kappa B, and neutrophil infiltration
nNOS inhibition	7-nitroindazole (7-NI)	Burn and smoke inhalation	Sheep [60]	IV infusion, 24 h	Decreased levels of PARP, pro-inflammatory cytokine IL-8, neutrophil infiltration Improved airway obstruction Improved oxygenation
Combined nNOS and iNOS inhibition	7-NI→BBS-2	Smoke inhalation and bacterial instillation	Sheep [61]	IV infusion, 12 h of 7-NI followed by 12 h of BBS-2	Improved airway obstruction Improved pulmonary gas exchange Decreased pulmonary VEGF, PARP, 3-NT No change in pulmonary edema
	7-NI+BBS-2	Burn and smoke inhalation	Sheep [62]	IV infusion, combined	Improved pulmonary oxygenation and mechanics Decreased lung lymph flow Decreased pulmonary edema
Hydrogen sulfide	H_2_S	Smoke inhalation	Rat [67]	Aerosolized	Decreased MDA, NO, iNOS, and NF-kappa B levels Improved oxidative stress
	Sodium sulfide	Burn and smoke inhalation	Mouse [68]	Subcutaneous injection	Decreased mortality Decreased pro inflammatory IL-1 beta, increased anti-inflammatory IL-10 Improved pulmonary histopathology
	Sodium sulfide	Burn and smoke inhalation	Sheep [69]	Bolus and IV infusion, 24 h	Decreased mortality Improved pulmonary oxygenation and mechanics Decreased pulmonary edema Decreased protein oxidation
HMG-CoA reductase inhibition	Simvastatin	Burn and smoke inhalation	Rat [72]	Oral	Decreased iNOS Reduction of pulmonary apoptosis Improved pulmonary histopathology
Proton pump inhibition	Esomeprazole	Smoke inhalation	Mouse [73]	Oral	Decreased levels of iNOS Decreased fibrosis Decreased plasma levels of pro inflammatory cytokine TNF-alpha
Solid lipid nanoparticles	Carvacrol	Smoke inhalation	Rat [86]	Aerosolized	Improved histopathology, Decreased oxidative injury (although also seen in oxygen treated groups) No change to myeloperoxidase levels

### Stem cell therapy

Mesenchymal stem cells are multipotent progenitor cells that contain the ability to differentiate into multiple cell lines. This unique characteristic has introduced stem cell therapy as a popular potential therapeutic modality due to the ability of stem cells to regenerate and repair different types of tissue after injury [7]. Studies have suggested the regenerative potential of bone marrow-derived mesenchymal stem cells (BMSC) in acute lung injury models [8–11]. Zhu et al. translated this experimental design to a rat model of smoke inhalation injury wherein BMSC were administered via tail vein injection after exposure to smoke. Results were significant for attenuation of injury in rats injected with BMSC after smoke exposure, with the authors also noting significant improvement of pulmonary histopathology, pulmonary edema, reduction in lung injury score, promotion of angiogenesis through paracrine activity, upregulation of vascular endothelial growth factor (VEGF), and involvement of the Notch1 signaling pathway [12]. Zhu et al. also investigated BMSC in a larger animal model and used rabbits to study the specific interaction between BMSC and VEGF. They administered smoke inhalation injury followed by intravenous injection of BMSC and then collected lung tissue up to 6 h after injury. They noted a significant increase in VEGF in injured lung tissue with a decrease in the amount of peripheral VEGF in these injured animals, as well as significant improvement in pulmonary edema in treatment groups when compared to controls [7].

Liu et al. used a rat model of smoke inhalation injury followed by tail vein injection of BMSC and studied the effects seen 24 h after injury. These authors noted improved pulmonary edema as well as decreased pro-inflammatory cytokine IL-8 with increased anti-inflammatory cytokine IL-10 in treatment groups when compared to controls [13]. Both Zhu et al. and Chen et al. saw similar alterations in pro- and anti-inflammatory cytokines in the serum in a rabbit model of smoke inhalation injury where animals were treated with BMSC immediately after smoke injury. These animals were evaluated up to 6 h after injury and all elevations in inflammatory cytokines (i.e., TNF-alpha, IL-1 beta, and IL-6) were significantly decreased in treatment groups when compared to animals that received smoke inhalation injury without bone marrow stem cell therapy [14, 15]. Additionally, Zhu et al. saw significant improvement in pulmonary edema measured by wet-to-dry ratio in animals that received BMSC after smoke inhalation injury when compared to controls [15].

In an attempt to localize human bone marrow-derived mesenchymal stem cells (hBMSC) to a site of pulmonary injury, Song et al. used luciferase (LUC) and green fluorescent (GFP) tagged hBMSC in a mouse model of smoke inhalation injury [16]. The LUC-GFP-hBMSC cells were administered via tail vein 24 h after smoke inhalation injury and monitored using bioluminescent (BLI) imaging. BLI signals were initially noted in both injury and control models, but BLI signal became much stronger in the pulmonary tissue of the injured group 24 h after injury, while the control group had migration of BLI signals to the abdomen at the same time point [16]. The authors also analyzed pulmonary parenchymal injury and pulmonary edema using wet-to-dry ratio and found that mice injected with hMSC had significantly lower wet-to-dry ratio when compared to untreated mice [16]. In addition, mice treated with hMSC also had histopathological improvement, increased keratinocyte growth factor [17] and decreased TNF-alpha in bronchial alveolar lavage fluid (BALF) [16].

Cui et al. evaluated the pulmonary effect of amnion mesenchymal stem cells administered after smoke inhalation injury. Human amnion mesenchymal stem cells (hAMSC) were injected in rats via the tail vein after exposure to white smoke [18]. The degree of inhalation injury was subsequently analyzed using CT scan, wet-to-dry ratio, histopathology, and evaluation of blood gases. Western blot was used to analyze surfactant protein levels and Enzyme Linked Immunosorbent Assay (ELISA) was used to detect inflammatory cytokines in BALF, both of which play a role in inflammation and pulmonary immune response. The results demonstrated overall pulmonary improvement in rats treated with hAMSC [18]. The labeled hAMSC were observed primarily in lung tissue after smoke inhalation injury, and the treatment group had significant improvement in CT pathology and histopathological score 28 days after inhalation injury, as well as decreased wet-to-dry ratio, indicating improvement in pulmonary edema [18]. There was also overall improvement in lungs treated with hAMSC based on evaluation of decreased pulmonary fibrosis, improved PaO_2_/FiO_2_ ratio, increased surfactant protein expression, decreased wet-to-dry ratio, and decreased BALF inflammatory cytokines in rats that had been treated with hAMSC after smoke inhalation injury [18].

Ihara et al. investigated the effect of adipose-derived mesenchymal stem cells on pulmonary microvascular changes using a sheep model of smoke inhalation injury [19]. Sheep were exposed to cotton smoke and then divided into groups that either received adipose-derived stem cells or a control vehicle. Treatment groups were noted to have improved pulmonary function demonstrated by improved PaO_2_/FiO_2_ ratio, oxygenation index, and improved peak airway pressures. In addition, these animals also had improvement in the pulmonary microvascular hyperpermeability seen after smoke inhalation injury which was evident by decreased lung lymph flow and increased plasma protein levels when compared to control animals that did not receive stem cell therapy [19].

Although these findings suggest that stem cell treatment may reduce inflammation and parenchymal damage after smoke inhalation injury, further studies to identify exact mechanism of action, as well as mortality benefit and additional therapeutic potential, may be beneficial. The potential use of stem cells may also be limited by a lack of readily available sources and potential need for expansion, posing a future challenge to translational research and clinical applicability.

### Anticoagulation targeted therapy

A number of compounds have emerged as potential therapies for inhalation injury which focus on different aspects of the disease process, including inflammation and airway obstruction. The combination of fibrin, cellular debris, and mucin form obstructive casts after smoke inhalation injury, allowing for propagation of the already hypoxic state due to decreased ventilation and increased perfusion, or ventilation/perfusion mismatch [20]. Additionally, obstruction leads to hyperinflation of alveoli which release the inflammatory cytokine and neutrophil chemotactant IL-8 causing further inflammation, loss of pulmonary hypoxic vasoconstriction, and injury [21, 22]. Administration of antithrombin III (ATIII), heparin, tissue plasminogen activator (tPA), and a combination of the above have been studied in a limited number of inhalation and burn injury animal models [21]. Enkhbaatar et al. used an ovine model of burn and smoke inhalation injury to further explore these therapeutic options. Initially they administered aerosolized tissue plasminogen activator (tPA) to sheep after cutaneous burn and smoke inhalation injury and found improvement of pulmonary oxygenation, vascular leakage, pulmonary edema, and airway obstruction with a high dose (2 mg vs. 1 mg) of tPA [23]. Enkhbaatar et al. also administered aerosolized ATIII and heparin to sheep after a similar model of combination of cutaneous burn and smoke inhalation injury and again found significant reduction in airway casts and obstruction, as well as lung lymph flow (an indication of transvascular fluid due to increased vascular permeability and an indirect indicator of severity of lung injury), compared to uninjured controls [24]. In addition, the authors observed decreased peak airway pressure, improved arterial pH, higher PaO_2_/FiO_2_ ratios, and reduced lung edema in ATIII/heparin treatment groups when compared to animals that were not exposed to burn or smoke injury [24]. Similarly, when the administration method of ATIII from nebulized form was altered to intravenous form (while maintaining the administration of nebulized heparin), similar results were observed with a significant improvement in pulmonary gas exchange, airway obstruction, and lung water content in animals burned, exposed to smoke inhalation injury, and treated when compared to untreated controls [25]. Miller et al. performed a systematic review and summarized continued investigations using this sheep model of burn and inhalation that administered a combination of anticoagulant therapeutics via inhalation, injection, or a combination of the two [21]. Notably, most combination regimens resulted in improved oxygenation with others also finding improved pulmonary edema and lung lymph flow in treated animals when compared to controls [21].

Miller et al. investigated a similar therapeutic approach in a retrospective study with historical controls and analyzed the survival benefit in patients treated with nebulized heparin, N-acetylcysteine, and albuterol versus those treated with albuterol alone after smoke inhalation injury. They found a significantly improved lung injury severity score in the treatment group after day 2 of hospitalization with significantly improved overall pulmonary function and improved survival [22]. Treatment with nebulized heparin has demonstrated symptom-specific beneficial effects in patients with inhalation injury; however, no established clinical guidelines exist regarding the dose or treatment strategy, or address the potential to develop a coagulopathy or heparin-induced thrombocytopenia in this specific patient population.

### Selectin inhibition

Therapeutic options targeted to the inflammatory response that occurs in the lungs after inhalation injury have also emerged with one mechanism of action specifically focused on the antibody neutralization of selectins. These selectins are composed of proteins either located on endothelial cells (E-selectin and P-selectin) or expressed on leukocytes (L-selectins) [26]. Medications targeted at proteins used for leukocyte adhesion and signal transduction such as L-selectin have been used in an attempt to improve the negative inflammatory effects of increased vascular permeability and edema after smoke inhalation injury [27]. One group investigated the effects of L-selectin blockade using an antibody to leukocyte adhesion molecule 1–3 (LAM 1–3) that they administered both before [28] and after burn and inhalation injury in sheep [27]. Initially they administered LAM 2 h prior to burn and smoke inhalation and found improvement in the delayed onset microvascular permeability without significant improvement in oxygenation [28]. In hopes of utilizing this as a more therapeutic approach, Katahira et al. administered LCAM after burn and smoke inhalation injury in a similar ovine model. This antibody administration was followed by monitoring of pulmonary transvascular fluid, gas exchange, cardio and pulmonary hemodynamics, histological changes, wet-to-dry ratio, and changes to overall leukocyte numbers. They found significantly less pulmonary fluid, lung lymph flow, and systemic neutrophil count in treated animals, but found no statistically significant changes to the histological exam, PaO_2_/FiO_2_ ratio, or wet-to-dry ratio in treated groups when compared to controls [27]. This study confirmed the main therapeutic effect to be on transvascular fluid flux and edema formation [27]. Chandra et al. focused on P-selectin blockade in a similar sheep model of burn and inhalation injury and found no difference in PaO_2_/FiO_2_ ratio or lung lymph flow in treatment groups when compared to control animals [26]. Chemokines and interleukins play a significant role in both lung injury and recovery and remain the subject of ongoing investigation after burn inhalation injury [29]. However, the results of these studies continue to vary due to changes in antibody administration time, dosage, or specific pulmonary benefit and require additional studies to evaluate these limitations, as well as overall mortality benefit in the study population. Additionally, administration of these experimental therapeutics provided systemic effects which could potentially limit clinical use and suggest the continued need for a pulmonary-specific therapeutic to target and treat smoke inhalation injury.

### Inflammatory pathway modulation

The pathological response to inhalation injury involves many aspects of the inflammatory cascade. In previous work from one of our labs, Dunn et al. developed a mouse model of inhalation injury that successfully recapitulated human injury, and in doing so noted an elevation in specific cytokines and damage associated molecular patterns (DAMPs) [30]. In particular, this project focused on the elevation of a neutrophil chemotactant CXCL1. The authors identified an attenuated response to pulmonary neutrophil infiltration after smoke inhalation injury by administering a neutralizing antibody to CXCL1, and also found a significantly decreased amount of inflammatory cytokines (IL-6, IL-1 beta, IL-16) in the treatment group compared to untreated controls [30]. It was also noted that lung histopathology and overall bacterial clearance improved in the group receiving the CXCL1 neutralizing antibody [30]. They hypothesized that blocking this early chemotactant attenuated early neutrophil recruitment, which ultimately prevented early lung damage and provided improved bacterial clearance after smoke inhalation injury [30].

Zhang et al. also investigated pulmonary inflammation after smoke inhalation injury using a rat model exposed to gun smoke. This study administered puerarin, a Chinese medicine with immunomodulatory properties, to rats intraperitoneally after smoke inhalation injury and found decreased neutrophils, vascular permeability, pulmonary edema, and cellular infiltrate in treatment groups after injury when compared to controls. They also noted increased levels of anti-inflammatory T cells (Th1) and a decrease in levels of Th2 which are thought to exacerbate acute lung injury through pro inflammatory signaling [31]. Ding et al. performed a randomized control trial that also focused on cytokines and inflammatory cells after smoke inhalation injury; however, this study used human subjects. The therapeutic investigation in this study centered on the administration of intratracheal perfluorohexane [32]. Perfluorohexane belongs to the perfluorocarbon family of chemical compounds which are liquids that have a low surface tension and high oxygen dissolving and carrying capacity. It has been utilized as a form of liquid ventilation to improve pulmonary gas exchange in a variety of models of pulmonary injury [33], but was noted to have significant systemic effects when administered intravenously [34, 35]. Ultimately, the authors found decreased numbers of neutrophils, IL-6, and IL-8 in bronchoalveolar lavage fluid after 3 days of treatment in the smoke inhalation injury group, but did not note any significant changes to systemic inflammation in the same group [32]. They also noted improvement in lung compliance after perfluorohexane therapy but were unable to detect significant differences in PaO_2_/FiO_2_ ratio in control vs. smoke inhalation injury patients [32]. Although the administration of vaporized intratracheal perfluorohexane was noted to decrease local inflammation, the long-term systemic effects remain unknown. Intravenous administration of perfluorocarbons have been noted to cause pneumothorax, lactic acidosis, and hypercapnia, which would provide significant clinical limitations if similar results were noted with intratracheal administration, as well as provide challenges related to production and storage of the compound, which would limit clinical benefit and translatability [34, 35].

Zhang et al. investigated the effect of suppressing cytokine signaling 1 (SOCS-1) on pulmonary inflammation and apoptosis using a mouse model of smoke inhalation injury [36, 37]. SOCS-1 downregulates pro inflammatory cytokines and stimulates cytokine signaling by activating the ubiquitin–proteasome pathway [36–38]. It also plays a role in macrophage activation and cytokine release leading to uninhibited and fatal inflammation in knockout animals [39]. SOCS-1 has been previously investigated in other models of acute lung injury and has been effective in regulating the inflammatory response and limiting pulmonary injury in these models [40, 41]. Zhang et al. used a mouse model of smoke inhalation injury to further investigate these protective effects. They administered intratracheal SOCS-1 prior to smoke inhalation injury and then monitored mice for up to 8 days. Treatment groups demonstrated improved mortality, healthier lungs on gross investigation, improved histopathology, decreased monocyte and neutrophil infiltration, and lower levels of pro inflammatory IL-1 beta and decreased caspase 1 cleavage when compared to controls [37].

Glutamine is utilized by critically injured tissue and known to be a conditionally essential amino acid. Previous studies investigating the role of glutamine in animal models of sepsis and acute lung injury have noted a promising benefit to lung tissue after injury [42, 43]. Li et al. administered glutamine via tail vein injection to rats after smoke inhalation injury. They found that treatment groups had improved histopathology, decreased edema, improved oxygenation, decreased levels of pro inflammatory cytokines, and reduced fibrosis. Interestingly, they also noted increased levels of protectant heat shock proteins in rats that had been treated with glutamine after exposure to smoke [44].

Administration of therapies focused on immunomodulation continues to be a subject of investigation in an emerging field using both animal and human studies of smoke inhalation injury, but continued experiments to identify systemic side effects, direct pulmonary therapeutic benefit, and overall mortality benefit would provide valuable information in this class of medications.

### Role of nitric oxide

The role of nitric oxide (NO) in burn and smoke inhalation injury has been investigated in multiple studies over the past decade using an ovine model of both cutaneous burn and smoke inhalation injury. Increased NO production is thought to be involved in the mechanism and exacerbation of pulmonary injury after burn injury through both loss of pulmonary hypoxic vasoconstriction and increased free radical formation through the interaction with superoxide and peroxynitrite formation. Under injured conditions pulmonary tissue experiences hypoxic vasoconstriction to preserve appropriate ventilation (V) and perfusion (Q) to better optimize gas exchange. Loss of this vasoconstriction by increased NO production results in blood flowing past unventilated alveoli, resulting in a V/Q mismatch, a pulmonary shunt, and tissue hypoxia [2, 45]. Additionally, elevated levels of NO react with superoxide to form peroxynitrite, which in turn causes DNA damage, ultimately leading to over-activation of DNA repair enzymes (poly(ADP-ribose)polymerase 1 or PARP) and induction of cellular stress, with the likely activation of NF-kappa B and accumulation of inflammatory cytokines [45–49].

Nitric oxide synthase (NOS), the source of nitric oxide, is expressed in multiple isoforms in the lung including endothelial (eNOS), neuronal (nNOS), and inducible NOS (iNOS). These isoforms are expressed in healthy lung tissue but are thought to have increased expression after burn and smoke inhalation injury. Cox et al. further investigated the distribution of NOS isoforms after burn and smoke inhalation injury using immunohistochemical analysis of lung tissue in a bovine animal model. They found that all three isoforms were expressed in uninjured pulmonary and bronchial tissue, but observed a significant increase in levels of iNOS in injury models [46].

### Superoxide and peroxynitrite decomposition

In addition to neutrophil activation, increased vascular permeability, pulmonary edema, and increased cytokine signaling and activation, oxygen free radicals contribute to the pulmonary pathological insult resulting from smoke inhalation injury [50]. Superoxide dismutase (SOD), which catalyzes the disassociation of superoxide into peroxide and oxygen, has been investigated for its potential therapeutic benefit after administration in smoke inhalation injury using sheep models but with limited success [51]. Bone et al. administered intravenous SOD to sheep at three different and increasing doses 1 h after smoke inhalation injury. They found no difference in hemodynamics, PaO_2_/FiO_2_ ratio, oxygen delivery, or lung lymph flow in treatment groups vs. control population, with the only significant change being increased oxygen consumption in the medium dose treatment group 24 h after smoke inhalation injury [51]. Additional studies investigated the benefit of administration of SOD after inhalation injury; however, these used nebulized SOD for direct intra-tracheal delivery. Maybauer et al. administered nebulized SOD to sheep at 1 h and 12 h after inhalation injury but again found no statistically significant difference in lung lymph flow, PaO_2_/FiO_2_ ratio, or pulmonary edema in treatment groups versus control [52]. Although nebulized SOD did not improve pulmonary oxygenation, gas exchange, or edema, treatment groups did have significantly lower levels of markers of lipid peroxidation and tissue injury (conjugated dienes) when compared to controls, suggesting an improvement in overall parenchymal injury in those animals treated with SOD, as well as lower levels of vascular leakage and protein loss [52]. These results differed from older studies that administered SOD prior to inhalation burn injury [53]; however, the recent studies utilized more clinically relevant animal models. The lack of a therapeutic benefit is thought to be secondary to the formation of superoxide radicals prior to administration of SOD, leading to irreversible free radical damage before potential beneficial effects of SOD [51, 52].

Understanding that superoxide reacts with NO to produce peroxynitrite, Hamahata et al. administered W-85, a peroxynitrite decomposition catalyst, to a similar ovine model of burn and smoke inhalation injury via direct cannulation of the bronchial artery. They found significant improvement in pulmonary oxygenation, shunting, and pulmonary vascular permeability in treatment groups when compared to uninjured controls, providing further confirmation of the role of NO in pulmonary injury after burn and smoke inhalation injury and the benefit of a therapeutic strategy aimed at peroxynitrite [54]. Similarly, Lange et al. administered INO-4885, a peroxynitrite decomposition catalyst, to sheep after burn and smoke inhalation injury for 24 h, initially given as a bolus dose followed by continuous infusion [55]. The study noted significant improvement in PaO_2_/FiO_2_ ratio, lung lymph flow, pulmonary edema, and airway pressures with a significant decline in the pro inflammatory cytokine IL-8 [55]. They also noted a significant decrease in VEGF and PARP, which may imply lower levels of cellular damage and oxidative stress, but did not note any benefits to survival and lacked a specific dose response study [55].

Ito et al. developed a combined model of injury using an ovine model of smoke inhalation injury and *Pseudomonas aeruginosa* infection to focus on the effect of superoxide degradation and peroxynitrite inhibition on pulmonary injury [56]. They administered an intravenous bolus followed by continuous infusion of R-100, a molecule that delivers NO while simultaneously facilitating the catalytic degradation of superoxide and hydrogen peroxide, for 24 h after inhalation injury. Treatment groups had significantly higher PaO_2_/FiO_2_ ratio with lower oxygen requirements, peak airway pressures, and overall fluid balance, all while maintaining similar cardiopulmonary hemodynamics when compared to controls [56]. There was no significant difference in histopathological appearance of lung tissue or wet-to-dry ratios when comparing these groups, but the treatment group had a trend towards pulmonary improvement [56]. No findings regarding mortality, nitrate or nitrite levels, or oxidative stress were noted in this study [56]. Enkhbaatar et al. described the administration of a non-selective NOS inhibitor, L-NG-nitroarginine methyl ester, which attenuated the negative effect on pulmonary gas exchange but also led to an increase in mean arterial and pulmonary arterial pressure, as well as a decrease in cardiac output [57]. This finding directed subsequent studies that focused on specific nitric oxide synthase inhibition to ameliorate lung injury after smoke inhalation with or without burn injury.

### Selective NOS inhibition

Specialized studies investigated the effects of iNOS inhibition in a sheep model of burn and inhalation injury. Enkhbaatar et al. administered BBS-2, an iNOS dimerization inhibitor, for 24 or 48 h after burn and inhalation injury via an intravenous approach to sheep [48, 57]. They first administered this therapy over a 48 h period and noted a delay in the decrease in PaO_2_/FiO_2_ ratio in treatment groups when compared to controls, implying a delay in the pulmonary injury and resultant inhibition of gas exchange seen after smoke inhalation injury [57]. In addition, treatment groups had significant improvement in lung lymph flow, pulmonary edema (measured by wet-to-dry ratio), and airway pressures with histological improvement in airway cast formation [57]. In an attempt to better characterize the mechanism of action of iNOS inhibition on amelioration of lung injury, Enkhbaatar later repeated this experiment but administered the therapy, BBS-2, for only 24 h as opposed to the original 48 h. Again, they noted similar improvements in the treatment animals, as well as significantly reduced levels of MPO, PARP, and pro inflammatory cytokine IL-8 in treatment groups when compared to controls [48]. Similarly, Pandareesh et al. noted decreased levels of iNOS after oral administration of *Bacopa monniera* (BME), a naturally occurring antioxidant and free radical scavenger, in rat models of smoke inhalation injury [58, 59]. This study investigated multiple pathways of inflammatory injury involving reactive oxygen species, lipid peroxidation, nitrite activity, acetylcholine esterase activity, GABA activity, and heat shock protein activity. Specifically, they found a significant decrease in iNOS levels that responded to oral BME administration in a dose dependent fashion, which may have contributed to improved pulmonary histopathology in treatment groups when compared to sham and control animals [58]. The inhibition of iNOS by BBS-2 and BME seemed to have positive effects on the pulmonary injury after smoke inhalation injury without notable negative systemic or cardiopulmonary sequelae during the study period [57, 58]. Further studies are needed to confirm the efficacy and mortality benefit of this therapeutic strategy.

Saunders et al. focused on the therapeutic effects of selective nNOS inhibition in a similar ovine model of pulmonary injury. In this study, 7-nitroindizole (7-NI), a specific nNOS inhibitor, was administered to sheep after burn and smoke inhalation injury via continuous intravenous infusion for 23 h after injury. It was noted that total NOS activity was higher in injured animals when compared to controls, and levels of NOS in treatment groups nearly returned to values similar to sham animals [60]. Additionally, other markers of injury such as lung tissue malondialdehyde, IL-8, histopathology score, airway pressures, and myeloperoxidase (MPO) activity were significantly lower in treatment groups, implying amelioration of the pulmonary injury that occurred after burn and smoke inhalation injury [60].

Lange et al. combined both of these treatment strategies in an animal model of smoke inhalation injury and sepsis. This study induced smoke inhalation injury to sheep followed by intratracheal installation of *P. aeruginosa*, then administered 7-NI for 1 to 12 h after injury and BBS-2 for 12 to 24 h after injury. This therapeutic approach was aimed at inhibition of both nNOS (via 7-NI) and iNOS (via BBS-2). Therapeutic groups had improvement in PaO_2_/FiO_2_ ratio, airway pressures, and histopathology scores but did not show any improvement in MPO activity (i.e., pulmonary neutrophil accumulation), tracheal blood flow, or pulmonary edema when compared to controls [61]. This therapeutic combination did not prove to be more efficacious than administering each treatment alone [61]. The same group then performed additional studies with the identical drug combination utilizing an ovine model of burn and smoke inhalation injury followed by a constant and simultaneous infusion of both 7-NI and BBS-2 for 48 h post-injury at a decreased dose when compared to previous experiments. In this scenario, they noted improvement in PaO_2_/FiO_2_ ratio, pulmonary shunting, and ventilator pressures, as well as lung lymph flow and complete prevention of pulmonary edema in treatment groups when compared to controls [62]. Notable limitations include varying infusion times between studies, optimal dosage in a larger patient population, and early termination of the study at 24 or 48 h. This short therapeutic course does not allow for evaluation of long-term systemic effects and does not provide information regarding overall mortality benefit. This simultaneous therapeutic approach proved more efficacious than previously noted, but further studies are needed to confirm the benefit of different levels of NOS inhibition in combination or alone.

### Hydrogen sulfide

Hydrogen sulfide (H_2_S) administration has been explored as another potential therapeutic for smoke inhalation injury due to its reported anti-inflammatory effects. Previously, these effects have been demonstrated in models of acute lung injury with notable decreases of pro inflammatory cytokines (IL-6, IL-8) and an increase in anti-inflammatory cytokine IL-10 in treatment groups [63]. In addition, administration of H_2_S was noted to inhibit cerebral peroxynitrite [64] and interfere with leukocyte infiltration and acute inflammation in vivo [65] as well as inhibit kappa B and subsequent iNOS expression in vitro [66]. Recently, Han et al. investigated the effect of aerosolized inhaled H_2_S on iNOS in vivo in a rat model of smoke inhalation injury and similarly noted a significant decrease in nitric oxide, iNOS, and NF-kappa B in H_2_S treatment groups when compared to smoke injured rats that were not treated with H_2_S [67].

Esechie et al. investigated the effects of administration of hydrogen sulfide in animal models of combined burn and smoke injury. Initially they applied this therapeutic strategy to a mouse model of burn and smoke inhalation injury, after which they administered sodium sulfide, a hydrogen sulfide donor, via a subcutaneous approach. Treatment groups had significant reduction in pro inflammatory IL-1 beta, elevated levels of anti-inflammatory IL-10, attenuation of histopathological injury of pulmonary tissue, as well as significantly decreased overall mortality [68]. This therapeutic strategy was then tested in an ovine model of burn and smoke inhalation injury, after which sodium sulfide was administered intravenously with a bolus dose followed by continuous infusion for 24 h post-injury. Exact levels of H_2_S were not directly measured. Again, Esechie et al. noted improved overall mortality in treatment groups, as well as a significantly increased PaO_2_/FiO_2_ ratio, pulmonary shunting, and airway pressures when compared to controls, without a significant improvement in neutrophil infiltration or pulmonary edema [69]. They also reported a significant decrease in iNOS expression, peroxynitrite formation, and PARP levels in treatment groups when compared to controls [69]. Although these data suggest attenuation of the resultant decline in pulmonary function after smoke inhalation injury by H_2_S administration, further studies focusing on dosage, microvascular or systemic vasodilation, and long term effects would prove beneficial. While H_2_S has been studied in other models of inflammation and lung injury, the exact mechanism of action after smoke inhalation injury remains unknown and would require further investigation before translating to a clinical model.

### HMG-CoA reductase inhibition

3-Hydroxy-3-methylglutaryl coenzyme A (HMG-CoA) reductase inhibitors describe the mechanism of action in a medication class known as statins. Statins are most known for their lipid-lowering action and are commonly used in patients with cardiogenic disease. Administration of these medications have also been reported to result in immunomodulatory and anti-inflammatory effects including decreased proinflammatory transcription factors and enzymes, adhesion molecules, and chemoattractant proteins [70, 71]. Based on this knowledge, Belli et al. administered oral statins to rats after burn and smoke inhalation injury to identify any possible protective effect on pulmonary injury [72]. They found significant improvement in histopathological scores, leukocyte infiltration and pneumocyte apoptosis in treatment groups when compared to controls, but did not note a significant difference in MPO levels between groups [72]. Levels of iNOS were significantly lower in treatment groups when compared to controls but not when compared to sham animals, and lipid peroxidation levels (measured by lung MDA) were significantly lower in treatment groups when compared to both control and sham [72]. In addition, they found significantly higher levels of glutathione concentrations in treatment groups when compared to both sham and control injury, implying protection against lipid peroxidation, oxidative stress, and apoptosis in injured mice treated with statins [72]. The application of this therapeutic may be limited by systemic effects of the drug. Oral administration limits pulmonary-specific benefits and further studies investigating the lipid lowering and systemic antioxidant effects in smoke inhalation injury patients would improve clinical translatability.

### Proton pump inhibition

Proton pump inhibitors (PPI) have been utilized in the gastrointestinal field as therapy for gastric acid overproduction and the associated conditions, but recent studies have emerged that investigate the extra-gastrointestinal effects of this class of medication due to previously noted anti-inflammatory and anti-fibrotic effects, as well as inhibition of iNOS expression [73, 74]. Nelson et al. investigated the effect of the PPI esomeprazole on mice after smoke inhalation injury. They administered oral esomeprazole at either 2 days post inhalation injury (preventative group) or 10 days post injury (treatment group) at either a high dose (300 mg/kg) or low dose (30 mg/kg). Interestingly, they found decreased levels of NO and iNOS in animals treated with the low dose PPI, as well as decreased plasma levels of inflammatory cytokine TNF-alpha and improved histopathology and fibrosis [73]. Although promising, the effects of this therapeutic are limited by oral administration. Further studies may also consider investigating the potential side effects and organ toxicity of this potentially dose dependent therapeutic prior to clinical use in patients with smoke inhalation injury.

### Targeted nanotherapies

Emerging therapies for smoke inhalation injury have proven to be a subject of current and upcoming research in a variety of animal models. The majority of the research reviewed above has utilized systemic administration of a medication while monitoring the effects on pulmonary injury. Therapeutic advances have also been made in a variety of pulmonary pathology, with most recent research focusing on targeted drug delivery via nanoparticles. Nanoparticles may be delivered via oral, transdermal, aerosol, or intravascular route and aim to target the site of pulmonary injury while avoiding possible systemic toxicity [75]. Specifically, inhaled nanotherapy uses aerosolized nanoparticles to deliver a therapeutic directly to injury site to maximize absorption and ease of administration while avoiding systemic effects or loss of medication through universal metabolization [76, 77]. Direct pulmonary delivery also provides a large surface area for medication absorption via alveolar epithelial cells, and size variations in specific nanoparticles can allow for retention in pulmonary alveoli if indicated [75]. Although promising, remaining concerns include potential lung toxicity, decreased absorption by injured lungs, potential removal by lung defense mechanisms, or variable drug stability in aerosolized form [77]. Studies have investigated the effect of inhaled nanotherapy in inflammatory disease [78] pulmonary hypertension [79, 80,] lung infection [81] and lung cancer [82–85] and the potential benefit of nanotherapy drug administration may be translatable to a model of smoke inhalation injury to improve therapeutic specificity. Recently, Carvalho et al. administered carvacrol, a natural oxyreducing compound, to the lungs after smoke inhalation injury in rats using a solid lipid nanoparticle (SLN) [86]. They noted improved histopathology in animals treated with carvacrol when compared to controls. In addition, they noted significant reduction of malondialdehyde (and therefore reduced levels of oxidative stress) but observed this change in both animals treated with oxygen and in animals treated with carvacrol when compared to animals exposed to smoke inhalation injury alone. There were no significant differences in inflammatory cells in treatment groups when compared to controls [86]. Multiple studies have also investigated the potential of lung targeted nanoparticle drug delivery after intravascular injection and found accumulation of nanoparticles in lung, liver, and spleen, although levels remained relatively low when compared to other organs in the reticuloendothelial system [75]. At this time, injectable nanoparticles have not been tested in a model of smoke inhalation injury. A specific targeted drug delivery system utilizing nanoparticle administration in combination with these emerging therapeutics may expand the treatment potential in patients with smoke inhalation injury and should serve as a future subject of investigation in this clinical scenario.

## Conclusions

There are a variety of emerging therapeutic options currently under investigation to treat smoke inhalation injury with or without burn injury. These treatment modalities have been tested in an assortment of animal models which have identified different mechanistic approaches to alleviate pulmonary injury resulting from smoke inhalation. While there appears to be promising results in all therapeutic categories, ongoing studies focusing on efficacy and mortality, as well as a longer treatment duration, would provide important information prior to translating these therapeutic options to a human study.

## Data Availability

All data analyzed during this study are included in this published article [and its additional files].

## Abbreviations

NOS: Nitric oxide synthase
eNOS: Endothelial nitric oxide synthase
nNOS: Neuronal nitric oxide synthase
iNOS: Inducible nitric oxide synthase
NO: Nitric oxide
PaO_2_/FiO_2_: Ratios of arterial partial pressure to inspired fraction of O_2_
FDA: US Food and Drug Administration
BMSC: Bone marrow-derived mesenchymal stem cells
VEGF: Vascular endothelial growth factor
hBMSC: Human bone marrow-derived mesenchymal stem cells
hAMSC: Human amnion mesenchymal stem cells
LUC- GFP: Luciferase and green fluorescent
BLI: Bioluminescent imaging
BALF: Bronchial alveolar lavage fluid
LISA: Enzyme Linked Immunosorbent Assay
ATIII: Antithrombin III
tPA: Tissue plasminogen activator
LAM: Leukocyte adhesion molecule
DAMPs: Damage associated molecular patterns
SOCS-1: Suppressing cytokine signaling 1
V: Ventilation
Q: Perfusion
PARP: Poly(ADP-ribose)polymerase 1
SOD: Superoxide dismutase
BME: *Bacopa monniera*
7-NI: 7-Nitroindizole
MPO: Myeloperoxidase
H_2_S: Hydrogen sulfide
HMG-CoA: 3-Hydroxy-3-methylglutaryl coenzyme A
PPI: Proton pump inhibitors
SLN: Solid lipid nanoparticle
